# Assessment of Factors Affecting Quality of Life in Oral Squamous Cell Carcinoma Patients Using University of Washington Quality of Life Questionnaire

**DOI:** 10.7759/cureus.3904

**Published:** 2019-01-16

**Authors:** Syed Abbas, Muhammad Usman U Tariq, Ahmed Raheem, Javeria Saeed, Syed S Hashmi, Musa Karim, Mazhar Nizam

**Affiliations:** 1 Otolaryngology, Aga Khan University Hospital, Karachi, PAK; 2 Pathology, Aga Khan University Hospital, Karachi, PAK; 3 Otolaryngology, Patel Hospital, Karachi, PAK; 4 Otolaryngology, Baqai Medical University, Karachi, PAK; 5 Miscellaneous, National Institute of Cardiovascular Diseases, Karachi, PAK; 6 Plastic Surgery, Patel Hospital, Karachi, PAK

**Keywords:** oral, squamous cell carcinoma, post-treatment, quality of life, score

## Abstract

Introduction

Post-treatment Quality of Life (QOL) is considered an important outcome in cancer patients. A number of questionnaire tools have been designed for its assessment. University of Washington Quality of Life (UW QOL) questionnaire version four is a reliable tool for assessment of post-treatment QOL in oral squamous cell carcinoma (OSCC) patients. Our aim was to identify the post-treatment problems faced by OSCC patients and to assess the impact of clinical factors affecting post-treatment QOL by using UW QOL (version four) questionnaire.

Methods

The study was conducted on 59 patients with OSCC who were treated with curative intent at Patel Hospital, Karachi from August 2015 to September 2015. Patients were asked to fill the UW QOL questionnaire (version four) on their follow-up visit.

Results

Overall mean composite QOL score was 66.59 ± 16.98. Chewing and saliva (dryness of mouth) had the lowest scores (38.98 ± 37.2 and 56.78 ± 41.4, respectively) among all domains while pain and anxiety had the highest scores (80.93 ± 20.4 and 79.66 ± 29.8, respectively). Patients having tumors of the tongue, late stage (III and IV) tumors, and restricted mouth opening had significantly lower mean composite QOL scores. Patients with tongue tumors revealed significantly lower scores for pain, swallowing, mood, and anxiety. Patients with late-stage tumors showed significantly lower scores for chewing, swallowing, taste, saliva, appearance, anxiety, and recreation. Patients with restricted mouth opening had significantly lower scores for pain, speech, appearance, recreation, and anxiety domains.

Conclusion

Different clinical features have different impacts on QOL in terms of problems faced by the patients. Features having a significant effect should be identified, and measures focused on most relevant problems should be employed in order to improve the post-treatment QOL.

## Introduction

Oral squamous cell carcinoma (OSCC) is a leading cause of cancer-related morbidity and mortality with the global annual incidence approaching 500,000 [1]. Annual OSCC incidence in Pakistan is 14,000 and urban Karachi has one of the highest incidences of OSCC worldwide [2]. Approximately 50% of these patients present with advanced stage (III or IV) disease and this translates into poor outcomes in survival pattern and the five-year survival rate is 20% [3]. Those who survive have varying degrees of compromised quality of life (QOL) [4]. Radiation to head and neck region has long-term implications such as limited mouth opening and dryness in the oral cavity [5]. The combined effect is significantly compromised speech and swallowing ability culminating in poor nutritional status and inability to return to a fully functional lifestyle.

There is a dire need to understand the functional limitations of these patients as a result of the disease process and subsequent interventions [6]. Significant evidence exists in favor of QOL as a predictor of cancer survivorship among OSCC patients [7]. Hence, disease-free survival (DFS) and improved QOL are now considered as combined endpoints in these patients. The last decade has witnessed intense focus on OSCC survivors and much effort has been put to identify the factors affecting the QOL. This would help in implementing therapies and measures aimed at improving the outcome.

Various QOL questionnaires have been used to estimate the outcomes in OSCC. University of Washington Quality of Life (UW QOL) questionnaire version four is a simple, brief and well-validated questionnaire that is globally employed in OSCC patients [8]. The incorporation of two important psychosocial factors (mood and anxiety) has made it a comprehensive tool to assess the outcome [9].

There is a paucity of literature from South Asia regarding QOL where OSCC is virtually endemic. This particular study aims at addressing the factors affecting post-treatment QOL in OSCC patients from a tertiary care hospital of Karachi, Pakistan. So far, no study has evaluated post-treatment QOL using the UW QOL questionnaire in Pakistani population. Identification of such possible prognosticators may help us start specific therapies aimed at improving the outcomes in these patients.

## Materials and methods

This cross-sectional study was conducted at our Patel Hospital, Karachi from August 2015 to September 2015. This study was approved by the hospital’s ethics committee (approval number: 44). A total of 120 patients with biopsy-proven OSSC were treated at our department during this period. Patients who underwent surgical excision with or without radiotherapy and/or chemotherapy with curative intent were included in the study. Patients treated with palliative intent and those treated with chemotherapy alone or chemoradiotherapy without surgery were excluded. Fifty-nine patients were finally selected in the study.

UW QOL questionnaire (Version 4) was employed as the questionnaire tool which is a self-reporting 12 point scale addressing pain, appearance, activity, recreation, swallowing, chewing, speech, shoulder function, taste, saliva, mood, and anxiety [10]. It also takes into consideration the most important issues faced by the patient over the last seven days. The responses from patients were rated from 0 (worst) to 100 (best) and mean scores for an individual was calculated. Patients with a higher mean score were interpreted to have a good outcome in terms of QOL.

In addition, there are two questions which address the patient’s perception about his/her well-being. These are Health Related QOL (HR-QOL) and Global QOL (G-QOL). HR-QOL assesses the overall health perception of the patient over the last seven days, while G-QOL assesses the overall QOL of the patient over the last seven days. These responses are scored on a 6 point Likert scale (ranging from very poor to outstanding). This purpose of the study was explained to the patients during their follow-up visit. Written informed consent was obtained from the patients. They were asked to fill out the questionnaire in a separate room and they were helped in filling out their responses where required.

Demographic and treatment-related details were obtained from the head and neck cancer database maintained at our department. The UW-QOL questionnaire related data was entered and analyzed using IBM SPSS Statistics for Windows, Version 21.0. (IBM Corp., Armonk, NY, US). The descriptive statistics were calculated for demographic variables in terms of mean ± SD. Patients were divided into two age groups (≤47 years and >47 years) based on median age, i.e., 47 years. Frequency and percentages were calculated for all categorical variables. Overall mean composite scores of QOL, HR-QOL, and G-QOL were calculated by using multiple response analysis. Seven factors were assessed for their impact on QOL in our patients. These include patient’s age, pre-operative oral addiction habits, restricted mouth opening, tumor site, tumor stage, treatment modality and follow up duration. The two-tailed analysis was conducted by applying the Mann–Whitney U test and Kruskal Wallis test to check the significance of the difference in mean QOL scores of each domain in different groups. The *p*-value of less than 0.05 was considered significant.

## Results

**Figure 1:**
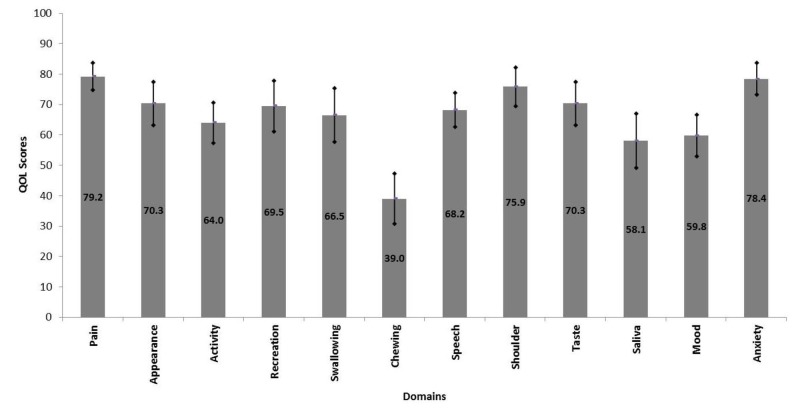
Mean quality of life scores of different domains in all patients.

Patient’s age ranged from 22 to 70 years with a mean ± SD of 47.9 ± 12.7 years. Male to female ratio was 1.36:1. The follow-up duration ranged from six to seventy-three months with a mean ± SD of 33.4 ± 19.6 months. Overall mean composite QOL score was 66.59 ± 16.98. Chewing and saliva had the lowest scores among all domains while pain and anxiety had the highest scores (Figure 1).

Patients having tumors of a tongue, late stage (III and IV) tumors, and restricted mouth opening had significantly lower mean composite QOL scores when compared with patients having tumors of cheek, early stage (I and II) tumors, and normal mouth opening, respectively. Females, older patients (> 47 years), patients without oral addiction habits and without co-morbid also had lower mean composite QOL scores when compared with patients males, older patients, (> 47 years), patients with oral addiction habits and with co-morbid, respectively. But the difference was statistically insignificant. Patients with single modality treatment had best mean composite QOL scores and there was a linear drop in QOL scores as the number of treatment modalities increased but statistical significance was not observed (Table 1).

**Table 1:** Comparison of Quality of Life Composite Score by Patient Characteristics

Characteristics	Frequency (%)	Composite Score [Mean ± SD]	p-value
Gender			
Male	34 (57.63%)	68.01 ± 16.71	0.438
Female	25 (42.37%)	64.67 ± 17.51	
Age			
Up to 47 years	30 (50.85%)	68.26 ± 11.98	0.676
More than 47 years	29 (49.15%)	64.87 ± 21.04	
Oral addiction habits			
Yes	46 (77.97%)	67.89 ± 16.81	0.309
No	13 (22.03%)	62.02 ± 17.48	
Co-morbids			
Yes	15 (25.42%)	70.42 ± 22.96	0.143
No	44 (74.58%)	65.29 ± 14.51	
Tumor site			
Tongue (anterior 2/3)	29 (49.15%)	62.14 ± 15.31	0.029*
Cheek	30 (50.85%)	70.9 ± 17.65	
Tumor stage			
Early Stage (I and II)	27 (45.76%)	74.38 ± 11.67	0.002*
Late Stage (III and IV)	32 (54.24%)	60.02 ± 18.11	
Mouth opening			
Within normal limits	17 (28.81%)	75.61 ± 15.93	0.003*
Restricted mouth opening	42 (71.19%)	62.95 ± 16.17	
Treatment modality			
Surgery	10 (16.95%)	71.46 ± 15.44	0.148
Surgery and radiotherapy	46 (77.97%)	66.8 ± 16.67	
Surgery, radio and chemotherapy	3 (5.08%)	47.22 ± 18.9	
Follow up duration			
Up to 1 year	8 (13.6%)	57.29 ± 19.2	0.244
1 to 2 years	17 (28.8%)	67.15 ± 15.99	
More than 2 years	34 (57.6%)	68.5 ± 16.7	

When patients with cheek tumors were compared with the patients having tongue tumors, domain-wise comparison revealed significantly lower QOL scores for pain, swallowing, mood, and anxiety (Table 2).

**Table 2:** Comparison of Quality of Life Domains by Tumor Site

Domain	Anterior 2/3 Tongue (n = 29) [Mean±SD]	Cheek (n = 30) [Mean±SD]	p-value
Pain	72.41 ± 13.93	85.83 ± 18.2	0.002*
Appearance	69.83 ± 27.04	70.83 ± 29.42	0.788
Activity	62.07 ± 25.55	65.83 ± 26.65	0.571
Recreation	66.38 ± 32.92	72.5 ± 32.4	0.436
Swallowing	58.62 ± 32.92	74.17 ± 34.42	0.029*
Chewing	39.66 ± 30.99	38.33 ± 33.95	0.826
Speech	62.93 ± 27.24	73.33 ± 14.58	0.137
Shoulder	72.41 ± 25.31	79.17 ± 24.64	0.236
Taste	65.52 ± 27.88	75 ± 27.07	0.122
Saliva	53.45 ± 33.22	62.5 ± 36.99	0.215
Mood	49.14 ± 24.53	70 ± 24.91	0.002*
Anxiety	73.28 ± 18.82	83.33 ± 21.1	0.019*

Patients with late-stage tumors showed significantly lower QOL scores for chewing, swallowing, taste, saliva, appearance, anxiety, and recreation, as compared to patients with early-stage tumors (Table 3).

**Table 3:** Comparison of Quality of Life Domains by Tumor Stage

Domain	Early Stage (n = 27) [Mean ± SD]	Late Stage (n = 33) [Mean ± SD]	p-value
Pain	82.41 ± 16.72	76.56 ± 17.89	0.206
Appearance	78.7 ± 26.59	63.28 ± 27.67	0.023*
Activity	68.52 ± 23.61	60.16 ± 27.58	0.221
Recreation	78.7 ± 31.55	61.72 ± 31.74	0.026*
Swallowing	83.33 ± 21.93	52.34 ± 36.68	0.001*
Chewing	50 ± 24.02	29.69 ± 35.6	0.008*
Speech	74.07 ± 12.94	63.28 ± 26.93	0.089
Shoulder	80.56 ± 20.02	71.88 ± 28.22	0.305
Taste	77.78 ± 25.32	64.06 ± 28.35	0.045*
Saliva	69.44 ± 29.69	48.44 ± 36.99	0.036*
Mood	64.81 ± 28.81	55.47 ± 24.37	0.149
Anxiety	84.26 ± 18.54	73.44 ± 21	0.037*

In comparison to patients with normal mouth opening, patients with restricted mouth opening had significantly lower scores for pain, speech, appearance, recreation, and anxiety domains (Table 4).

**Table 4:** Comparison of Quality of Life Domains by Status of Mouth Opening

Domain	Within Normal Limits (n = 17) [Mean ± SD]	Restricted Mouth Opening (n = 42) [Mean ± SD]	p-value
Pain	88.24 ± 15.61	75.6 ± 17.01	0.01*
Appearance	86.76 ± 17.94	63.69 ± 28.8	0.004*
Activity	69.12 ± 28.68	61.9 ± 24.84	0.385
Recreation	86.76 ± 25.18	62.5 ± 32.78	0.004*
Swallowing	72.06 ± 34.1	64.29 ± 34.55	0.374
Chewing	52.94 ± 37.38	33.33 ± 28.51	0.052
Speech	80.88 ± 20.78	63.1 ± 20.83	0.004*
Shoulder	80.88 ± 27.29	73.81 ± 24.04	0.152
Taste	64.71 ± 36.51	72.62 ± 23.3	0.670
Saliva	66.18 ± 37.44	54.76 ± 34.14	0.190
Mood	70.59 ± 25.36	55.36 ± 26.21	0.043*
Anxiety	88.24 ± 15.61	74.4 ± 21.02	0.013*

Interestingly, patients without pre-operative oral addiction habits showed significantly lower QOL scores for activity (48.08 ± 16.01 versus 72.28 ± 26.99) and recreation (57.69 ± 35.92 versus 78.26 ± 31.89) domains (*p*-value: 0.004 and 0.044, respectively).

When assessed for effect of the number of treatment modalities received by the patients, there was a decrease in mean scores of almost all domains but statistical significance was observed for anxiety domain only (*p*-value: 0.004) (Table 5).

**Table 5:** Comparison of Quality of Life Domains by Treatment Modalities

Domain	Surgery Alone [n = 10] [Mean ± SD]	Surgery and Radiotherapy [n = 46] [Mean ± SD]	Surgery, Radio and Chemotherapy [n = 3] [Mean ± SD]	p-value
Pain	82.5 ± 16.87	78.8 ± 18.23	75 ± 0	0.764
Appearance	72.5 ± 21.89	71.2 ± 29.33	50 ± 25	0.439
Activity	67.5 ± 23.72	64.67 ± 26.65	41.67 ± 14.43	0.301
Recreation	55 ± 34.96	74.46 ± 30.95	41.67 ± 28.87	0.070
Swallowing	72.5 ± 34.26	66.85 ± 34.18	41.67 ± 38.19	0.397
Chewing	50 ± 33.33	38.04 ± 31.95	16.67 ± 28.87	0.271
Speech	72.5 ± 27.51	67.93 ± 21.51	58.33 ± 14.43	0.622
Shoulder	82.5 ± 20.58	76.09 ± 24.13	50 ± 43.3	0.141
Taste	85 ± 17.48	68.48 ± 27.61	50 ± 43.3	0.097
Saliva	75 ± 20.41	53.8 ± 37.63	66.67 ± 14.43	0.207
Mood	65 ± 29.34	60.33 ± 26.13	33.33 ± 14.43	0.189
Anxiety	77.5 ± 18.45	80.98 ± 17.63	41.67 ± 38.19	0.004*

We also assessed the effect of follow-up duration on QOL scores in different domains. We observed that the scores for chewing, swallowing, taste, and appearance were the lowest in patients with a follow-up duration of less than one year, while scores for speech, saliva, mood, and anxiety were highest in these patients. However, these differences in scores on the basis of follow-up duration were not statistically significant (Table 6).

**Table 6:** Comparison of Quality of Life Domains by Follow-up Duration

Domain	≤1 year [n = 9] [Mean ± SD]	>1-2 years [n = 16] [Mean ± SD]	>2 years [n = 34] [Mean ± SD]	p-value
Pain	80.56 ± 20.83	78.13 ± 20.16	83.82 ± 16.15	0.649
Appearance	58.33 ± 25	71.88 ± 25.62	79.41 ± 25.72	0.073
Activity	61.11 ± 22.05	73.44 ± 24.95	65.44 ± 28.88	0.482
Recreation	66.67 ± 33.07	79.69 ± 33.19	72.79 ± 34.47	0.436
Swallowing	58.33 ± 46.77	60.94 ± 41.8	71.32 ± 37.5	0.651
Chewing	22.22 ± 36.32	34.38 ± 35.21	45.59 ± 37.67	0.190
Speech	83.33 ± 21.65	68.75 ± 33.54	75 ± 26.83	0.565
Shoulder	69.44 ± 34.86	90.63 ± 15.48	77.21 ± 29.75	0.174
Taste	52.78 ± 42.29	73.44 ± 28.09	74.26 ± 35.08	0.254
Saliva	61.11 ± 37.73	53.13 ± 41.71	57.35 ± 43.31	0.899
Mood	69.44 ± 37.03	59.38 ± 30.1	64.71 ± 28.28	0.604
Anxiety	80.56 ± 32.54	79.69 ± 29.18	79.41 ± 30.45	0.987

When divided on the basis of patient’s age, no significant difference of QOL scores was observed for any domain.

**Figure 2:**
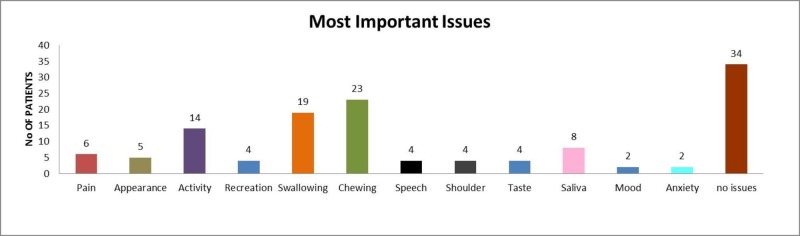
Most important issues experienced by patients in last seven days.

On HR-QOL and G-QOL scales, a majority of the patients responded as fair, good, very good or outstanding (88% and 91.5%, respectively). Chewing and swallowing were the two major problems faced by the patients in the last seven days. Mood and anxiety were the least frequently described issues in our study group. More than half (57.6%) of the patients did not experience any issues in the last seven days (Figure 2).

## Discussion

The assessment of QOL is a complex issue involving the evaluation of different domains such as speech, pain, chewing, etc. As the outcome entails multiple factors, alternative study designs could include multivariate assessments of QOL domains. The UW-QOL questionnaire has already been validated by studies comparing the results of its application with those obtained from other well-established questionnaires in the same field of study. Besides effectively assessing QOL, the UW-QOL was well accepted by respondents and appraised as a reliable, practical, and low-cost instrument for surveying the functional status of patients with head and neck cancer [11]. However, the feasibility of its use in different languages still demands further research.

The salient feature of this study is that it highlights the factors affecting the QOL in post-operative OSCC patients. Few studies have described QOL issues in OSCC patients as a select group. Most often oral QOL issues are brought to the limelight as a subgroup of diverse head and neck cancers. Subsequently, issues related to OSCC are overwhelmed by general issues faced by head and neck cancer patients.

Male predominance seen in the participants of this study is in concordance with existing studies [12-19]. Mean patient’s age in our study (47.9 ± 12.7 years) was younger than mean patients’ age reported by several studies [12-14,16,19]. Male patients were slightly elder (mean ± SD: 48.5 ± 12.2 years) than the female patients (mean ± SD: 45.4 ± 12.1 years) but this difference was statistically insignificant (p = 0.536).

The overall mean QOL score in our study was 68.2 ± 24.7 and it was lower than the mean QOL score (74.6 ± 18.2) of the study by Bhanja et al. [20]. Mean domain scores of each component were measured using UW-QOL scale and the problems related to chewing (38.98 ± 32.25), saliva (58.1 ± 35.18), and mood (59.8 ± 26.6) had minimum scores in our study. Similarly, in the study by Nagy et al. [14] and Andrade et al. [21], the major problem faced by the patients was also chewing. However, Rogers et al. [22] reported that 45% of patients in his study did not experience any chewing problem. Similarly, Bhanja et al. [20] also reported mood and anxiety as the major problems with mean values 46.5 ± 23.7 and 56.4 ± 26.9, respectively. These observations reinforce the importance of dental rehabilitation of patients subjected to surgical resection of OSCC. Speech impairment has been highlighted to be a concern for OSCC patients in some previous studies, but we observed good scores for speech in our study [11-14].

Overall mean composite QOL score of cheek tumor patients in our study (70.9 ± 17.65) was comparable to the mean QOL score of cheek tumor patients in the Bhanja et al. [20] study. Overall mean composite QOL score of patients with tongue tumors was significantly lower than scores of patients with cheek tumors in this study (p = 0.029). The difference in scores of pain, swallowing, mood, and anxiety domains between these two groups can be explained by increased innervation and the critical role of tongue in swallowing.

Problems like chewing, swallowing, taste, saliva, appearance were experienced to a greater extent in late-stage tumors and can be attributed to extensive surgery. In addition, these patients also receive adjuvant radiotherapy and/or chemotherapy which can cause fibrosis leading to difficulty in chewing, salivation, and disfigurement. Nausea, vomiting, weakness, and other side effects related to chemotherapy may also contribute to anxiety, mood disturbances, and lack of recreation in these patients receiving adjuvant treatment. This hypothesis is also favored by decreasing mean composite QOL scores with increasing modalities.

Patients with restricted mouth opening experienced more pain and difficulty in the speech which can be attributed to fibrosis and difficulty in mouth opening. Anxiety, lack of recreational activity, and the sense of poor appearance might partially be explained by the psychological impact of the disease.

Regarding HR-QOL and G-QOL, chewing, swallowing, activity, appearance, saliva, and pain were some of the important issues of patients in the last week before filling the questionnaire. These issues have also been highlighted in some of the previously reported studies [20-21].

Several studies have described the post-treatment evaluation of HR-QOL for OSCC patients and assessed factors associated with improvements in prognosis. These studies reinforce the hypothesis that patients who survived surgery may effectively improve and even recover their HR-QOL levels, at least to pre-operative ratings [11,14,19,23-24]. The improvement of certain QOL domain scores with increasing follow up duration, as seen in our study, points to the fact that cancer patients in the initial years of follow up experience psychological stress and post-treatment effects of different treatments they receive. These problems settle down with time and therefore, these patients need proper psychological counseling and support.

In the light of our findings, we think that patients with late-stage tumors and tongue tumors should be actively managed for pain. There should be special education and physical training for these patients to improve their mouth opening, chewing, speech, and swallowing. The use of liquid and semisolid diet in the initial post-operative period will also help in reducing these complaints. These patients should also get special counseling from a psychiatrist for their anxiety and mood problems.

Main limitations of this study are small sample size and single-center experience. In addition, self-reporting of the questionnaire by the patients bear the potential of over and/or underestimation. Multi-center studies on a larger cohort of patients are required to address the issues of postoperative QOL. Furthermore, prospective studies should be carried out to assess the role of interventions done to improve QOL.

## Conclusions

Post-treatment QOL assessment of OSCC patients reveals a number of problems such as chewing, swallowing, saliva, lack of activity, mood disturbances, etc. Pre-operative clinical features including tumor site, tumor stage, and extent of mouth opening have a significant impact on post-treatment problems in different ways. Proper and timely interventions help in achieving better QOL levels early and closer to pre-operative levels.
